# Metatranscriptomic analysis of an *in vitro* biofilm model reveals strain-specific interactions among multiple bacterial species

**DOI:** 10.1080/20002297.2019.1599670

**Published:** 2019-04-11

**Authors:** Yifei Zhang, Wenyu Shi, Yeqing Song, Jinfeng Wang

**Affiliations:** a Central Laboratory, Peking University School and Hospital of Stomatology & National Clinical Research Center for Oral Diseases & National Engineering Laboratory for Digital and Material Technology of Stomatology & Beijing Key Laboratory of Digital Stomatology, Beijing, China; b Beijing Institutes of Life Science, Chinese Academy of Sciences, Beijing, China

**Keywords:** Biofilm, periodontitis, periodontal microbiology, bacteria interaction, periodontal pathogen

## Abstract

Interactions among bacteria can affect biofilm properties.

**Method**: Here, we investigated the role of different bacteria in functional dysbiosis of an *in vitro* polymicrobial subgingival plaque model using both 16S rRNA and metatranscriptomic sequencing.

**Results**: We found that high-virulence *Porphyromonas gingivalis* W83 had greater effects on the symbiotic species than the low-virulence *P. gingivalis* ATCC33277, and that *Prevotella intermedia* exacerbated the effects of W83. *P. gingivalis* significantly influenced the expression of genes related to metabolic pathways and quorum sensing of commensal oral species in a strain-specific manner. *P. intermedia* exerted synergistic effects with *P. gingivalis* W83 but antagonistic effects with strain ATCC33277, which may regulate the expression of virulence factors of *P. gingivalis* through the clp regulator.

**Discussion**: The interaction networks indicated that the strongest correlation was between *Fusobacterium nucleatum* and *Streptococcus mitis*, which demonstrated their bridge and cornerstone roles in biofilm. Changes in the expression of genes relating to outer membrane proteins in *F. nucleatum* indicated that the addition of different bacteria can interfere with the co-adherence among *F. nucleatum* and other partners.

**Conclusion**: We report here the existence of strain-specific interactions in subgingival plaque, which may enhance our understanding of periodontal micro-ecology and facilitate the development of improved plaque control strategies.

Periodontitis is a highly prevalent polymicrobial infectious disease affecting the supporting tissues of the teeth and is associated with systemic diseases, such as diabetes, rheumatoid arthritis, and atherosclerosis [1–3]. Dental plaque is known to be the initial factor of periodontitis. There are several different hypotheses and ideas on the role of plaque in the development of periodontitis (reviewed in [4]). According to the ‘Specific Plaque Hypothesis’, only a few species in the resident plaque microbiota can cause disease. However, these putative pathogens can also be identified in healthy sites without initiating disease [5]. This led to the ‘Non-Specific Plaque Hypothesis’ being proposed, in which the bacteria would behave as integrated communities to cause disease, only some bacteria (perhaps specific) may be more virulent than others and be able to predominate. Further, Marsh proposed the ‘Ecological Plaque Hypothesis’ which combined the key concepts of the earlier two hypotheses [6]. It proposed that the dysbiosis of the subgingival microbiota is the major cause of periodontitis. Under normal circumstances, the dynamic balance between oral bacteria and the host maintains oral health. Disturbance of this balance would lead to disease. Studies have shown that certain clusters or complexes of bacteria are more closely associated with periodontitis [7]. The ‘red complex’ – *Porphyromonas gingivalis, Tannerella forsythus*, and *Treponema denticola* – is most frequently identified in deeper periodontal pockets and related to ‘chronic’ severe periodontitis. Their presence was preceded by members of the ‘orange complex’ (*Fusobacterium* spp., *Prevotella intermedia, Prevotella nigrescens, Peptostreptococcus micros, Streptococcus constellatus, Eubacterium nodatum, Campylobacter gracilis*, and *Campylobacter rectus*), which was also strongly associated with bleeding on probing and increased pocket depth. In contrast, members of the yellow (mainly *Streptococcus* spp.), green (*Capnocytophata* spp., *Campylobacter concisus, Eikenella corrodens*, and *Aggregatibacter actinomycetemcomitans* serotype a), blue (*Actinomyces* species) and purple (*Veillonella parvula* and *Actinomyces odontolyticus*) complexes are generally associated with healthy periodontal sites. Thus, periodontitis is a result from the activity of mixtures of interacting bacteria.

In the opinion of Hajishengallis, certain bacterial species play a critical role, which led to the ‘Keystone-Pathogen Hypothesis’ being proposed [8]. This hypothesis indicates that certain low-abundance microbial pathogens, for example, *P. gingivalis*, can initiate destructive inflammation by transforming a symbiotic into a dysbiotic microbiota, although *P. gingivalis* alone does not cause periodontitis in germ-free mice [9]. Although a number of microbiome studies have provided a wealth of information on the difference of periodontal microbial ecology between health and disease at the taxonomic or species level [10–13], the shifts from health to disease during the periodontitis process have not yet been explained well by current hypotheses.

On the other hand, the virulence of certain strains could differ among different individuals or even in different sample sites within the same person. For example, types of fimbriae, which are critical determinants of *P. gingivalis* virulence, differed between health and disease: in healthy adults, the most prevalent fimA genotype was type I (66.7%), while in the patient group type II was detected most frequently (43.6%), followed by type IV (30.9%) [14]. *P. intermedia* is a member of ‘orange complex’ that closely associated with periodontitis [7]. In our previous study, it was found that the clinical strains of *P. intermedia* isolated from diseased sites had more unique virulence factors and metabolic pathways than strains from healthy sites in the same cohort of subjects [15]. Since bacteria within a biofilm communicate through physical contact (coaggregation and coadhesion), as well as signal transduction (e.g., quorum sensing) and metabolic interactions [16], the interactions within different communities may affect the virulence of different bacterial strains [17,18]. Accordingly, the differences at strain-level rather than species level in the development of periodontitis should be investigated in more detail.

Here we established an *in vitro* model of subgingival plaque using five strains typically found in healthy periodontal sites. *P. gingivalis* ATCC33277 and W83 were added, respectively, with or without a *P. intermedia* isolate from a diseased periodontal site. *P. gingivalis* ATCC33277 is classified as avirulent/non-invasive, and W83 as virulent/invasive [19]. The effects of *P. gingivalis* strains with different levels of virulence on community dysbiosis were assessed, and the impact on the virulence of different *P. gingivalis* strains of the diseased-derived *P. intermedia* was investigated. We identified strain-specific symbiotic relationships among species and demonstrated the effects of *P. gingivalis* strains with different levels of virulence on commensal community dysbiosis (both structural and functional), and revealed the strain-specific impact of the *P. intermedia* on the virulence of *P. gingivalis*. We further proposed that the outer membrane proteins in ‘bridging’ (*F. nucleatum*) and the quorum sensing (QS) signal system in ‘keystone’ (*P. gingivalis*) may be crucial factors in mediating community interactions.

## Materials and methods

## Bacterial strains and culture conditions

*S. mitis* 86-5, *Actinomyces naeslundii* 86-2, *F. nucleatum* 86-6, *V. parvula* 86-3, and *C. gracilis* 86-9 were isolated from the same healthy site of a periodontitis patient; *P. nigrescens* 84-4 were isolated from a healthy periodontal site of the second patient, and *P. intermedia* 63-5 from a diseased periodontal site of the third patient [15]. The isolates were identified by full-length 16S rDNA sequencing (Sangon Biotech, Shanghai, China). *P. gingivalis* ATCC33277 and *P. gingivalis* W83 were purchased from the American Type Culture Collection (Rockville, MD). All strains were cultured to mid-log phase in tryptone soy broth (Oxoid, Hampshire, UK) supplemented with 5 µg/mL hemin and 1 µg/mL vitamin K under anaerobic conditions (90% N_2_, 5% H_2_, and 5% CO_2_) at 37°C.

## *In vitro* model of subgingival plaque

**Figure 1.**
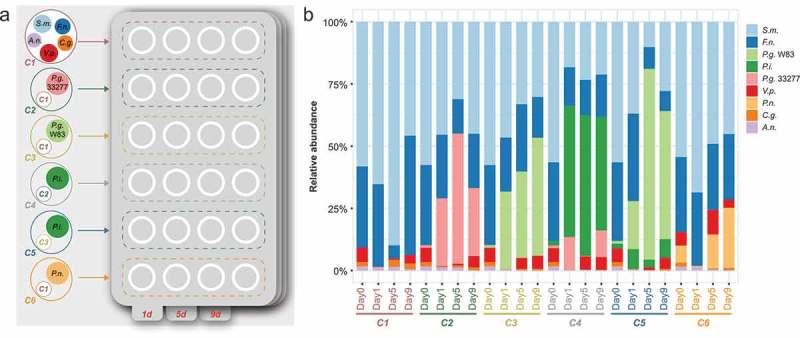
Dynamics of six bacterial communities of different compositions. (a) The diagram depicts the design of six bacterial communities (C1-C6) with different compositions; (b) The relative abundance of each strain in different communities (C1-C6) at different time points (0, 1^st^, 5^th^ and 9^th^ day) assessed by 16S rRNA sequencing analysis.

Community 1 (C1) (commensal microbiota) comprised *S. mitis, A. naeslundii, F. nucleatum, V. parvula*, and *C. gracilis*. To evaluate the impact of red complex members on the commensal microbiota, *P. gingivalis* strains ATCC33277 (low virulence) or W83 (high virulence) were introduced into C1, producing C2 and C3, respectively. Addition of a *P. intermedia* isolate from periodontal disease into C2 and C3 formed C4 and C5, respectively. As a control, a clinical *P. nigrescens* strain isolated from a healthy periodontal site was added to C1, forming C6 (Figure 1(a)). Biofilms were grown in 24-well culture plates on sterile hydroxyapatite discs (9.5 × 2 mm; Clarkson Chromatography Products Inc., South Williamsport, PA). Each well contained 1 mL of artificial gingival crevicular fluid (GCF) [20], which comprised 60% Roswell Park Memorial Institute medium (RPMI; Hyclone, GE Healthcare, Chicago, IL) and 40% defibrinated horse serum (Gibco, Thermo Fisher Scientific, Waltham, MA). To form the acquired pellicle, the discs were allowed to stay in contact with the medium for at least 24 h under anaerobic conditions prior to inoculation. Next, 2 mL of fresh GCF medium and 40 μL of a mixture of *S. mitis, A. naeslundii, F. nucleatum, V. parvula*, and *C. gracilis* (58:2:32:6:2%, around 8–9 × 10^8^ cfu/mL total) were added. To mimic a pathogenic biofilm, 5 μL of a *P. gingivalis* suspension (around 1.5–2.0 × 10^7^ cfu/mL) were added to C2 (ATCC33277) and C3 (W83). To evaluate the interactions among periodontal pathogens, 5 μL of a *P. gingivalis* ATCC33277 or W83 suspension plus 5 μL of a *P. intermedia* suspension (2.5 × 10^7^ cfu/mL) were added to C4 and C5. Five microliters of a *P. nigrescens* suspension (1 × 10^8^ cfu/mL) were added to C6 (control). The plates were incubated in an anaerobic chamber for 9 days. At 48-h intervals, 1 mL of culture fluid was aspirated from each well and replaced with 1 mL of medium. Biofilm-containing disks were removed at 1, 5, and 9 days after inoculation in quadruplicate.

## DNA and RNA extraction

Biofilm samples were transferred to screw-cap tubes containing 2 mL PBS and bead beaten (Biospec Mini-BeadBeater, USA) for 1 min at 4,200 rpm. Parallel samples were pooled to guarantee sufficient RNA yield and reduce intrasample deviation. Each pooled sample was split for DNA and RNA extraction. Bacteria were pelleted by centrifugation at 12,000 × *g* for 3 min. DNA and RNA were extracted following the protocol of the QIAamp DNA Mini Kit for DNA and the RNeasy Mini Kit for RNA (Qiagen, Hilden, Germany).

## 16S rRNA gene amplification, cloning, and sequencing

The V4–V5 hypervariable regions of the 16S rRNA gene were amplified using the primers 515F (5′-GTGCCAGCMGCCGCGG-3′) and 907R (5′-CCGTCAATTCMTTTRAGTTT-3′) []. PCR was performed in a 20 μL mixture containing 0.8 μL of each primer (5 μM), 10 ng template DNA, 2 μL 2.5 mM dNTPs, 0.4 μL FastPfu Polymerase, and 4 μL 5 x FastPfu Buffer. The PCR conditions were: 3 min at 95°C; followed by 27 cycles of 30 s at 95°C, 30 s at 55°C, and 45 s at 72°C; and 10 min at 72°C. Amplicons were resolved by electrophoresis in 2% agarose gels. Purified amplicons were pooled and paired-end sequenced (2 × 250) on an Illumina MiSeq platform (Illumina, San Diego, CA) according to standard protocols by Majorbio Bio-Pharm Technology Co. Ltd. (Shanghai, China).

## RNA sequencing

Samples 2, 3, 4, 5, and 6 at day 9 demonstrated the most stable patterns and were therefore subjected to total RNA sequencing. Prior to sequencing, RNA integrity was checked using a Bioanalyzer 2100 (Agilent Technologies, Santa Clara, CA). Qualified total RNA was purified using an RNeasy Micro Kit (Qiagen) and RNase-Free DNase Set (Qiagen). rRNA was removed using Ribo-Zero rRNA Removal Kits (Epicentre, Madison, WI). RNA-seq libraries were prepared using the TruSeq Stranded Total RNA LT Sample Prep Kit (Illumina) according to the manufacturer’s protocol. Libraries were quantified and analyzed using a Qubit 2.0 (Thermo Fisher Scientific) and a Bioanalyzer 2100 (Agilent). Paired-end 100 bp sequencing was conducted on an Illumina HiSeq 2500 system at Biotechnology Corporation (Shanghai, China).

## Processing of 16S rRNA sequencing data

Raw fastq files were demultiplexed, quality-filtered by Trimmomatic, and merged by FLASH with the following criteria: (i) Reads were truncated at any site with an average quality score of <20 over a 50-bp sliding window; (ii) two nucleotide mismatches were allowed in primer sequences and reads containing ambiguous bases were removed; and (iii) sequences with >10-bp overlaps were merged according to their overlap sequence. Databases were constructed using the full-length 16S rRNA sequences of reference strains (Table S7). The optimized sequences were aligned to the corresponding database using BLASTN. Hits were considered significant at an e-value ≤1e−05.

## Quantitative analysis of gene expression

The metatranscriptomic sequences of samples 2–6 were mapped to the corresponding database (Table S7) using bowtie2 (ver. 2–2.0.5) [21]. Gene fragments were counted using HTSeq [22] and normalized by the trimmed mean of M values (TMM) [23]. Gene expression levels are shown as fragments per kilobase of an exon model per million mapped reads (FPKM), as follows:

$$FPKM=\frac{total\;exon\;Fragments}{mapped\;reads\left(Millions\right)\times exon\;length\left(KB\right)\;}$$

## Statistical analysis of gene expression

Differences in gene expression were analyzed using edgeR [24], and *P*-values were adjusted using multiple hypothesis tests. The *P*-value threshold was determined by controlling the false discovery rate (FDR). The *q*-value was defined as the adjusted *P*-value. We calculated fold-changes in gene expression using the FPKM values. Differences were considered significant at a *q*-value ≤0.05 and fold-change ≥2.

## Gene functional categories

We mapped the DEGs to known biological ontologies based on KEGG orthology classifications (https://www.kegg.jp/kegg/).

## Inter-species networks

Correlations between genes were evaluated based on the gene expression levels at day 9 using SparCC with 100 bootstraps to estimate the *P*-values. Genes with correlation values >0.8 and *P*-values <0.05 were regarded as strongly correlated.

## Results

## Community structure dynamics of the biofilm model

We constructed six bacterial communities of different compositions (C1–C6; Figure 1(a)). We first assessed the community composition of six communities after 1, 5, and 9 days by 16S rRNA sequencing (Figure 1(b)). In comparison to C1, *P. nigrescens* did not cause significant alterations in community composition and structure at any time point (C6), while both *P. gingivalis* ATCC33277 (C2) and W83 (C3) caused shifts in the relative abundance of commensal species and significant alterations in community composition and structure, with *P. gingivalis* W83 causing a more severe dysbiosis than *P. gingivalis* ATCC33277: the percentage of members in the ‘yellow’, ‘blue’ and ‘purple’ complexes exhibited a steady decrease from the 1^st^ to the 9^th^ day, while the percentage of members in the ‘red’ (*P. gingivalis* W83) and ‘orange’ (mainly *F. nucleatum*) complexes exhibited a steady increase and made a dominating proportion (>60%) on the 5^th^ and 9^th^ day (C3). The relative abundance of each strain changing over time in different communities were shown in Figure S1. The presence of *P. intermedia* enhanced the effects of *P. gingivalis* ATCC33277 (C4) and *P. gingivalis* W83 (C5) on the community dysbiosis. The results revealed that *P. gingivalis*, and particularly strain W83, modulated the relative abundance of symbiotic species, whereas a non-virulent member of the orange complex (*P. nigrescens*) did not. However, the abundances of symbiotic species varied over time. We also found that *P. intermedia* enhanced the growth of *P. gingivalis* W83 but suppressed that of *P. gingivalis* ATCC33277.

## Functional changes of commensal strains in response to the interference of keystone pathogens

**Figure 2.**
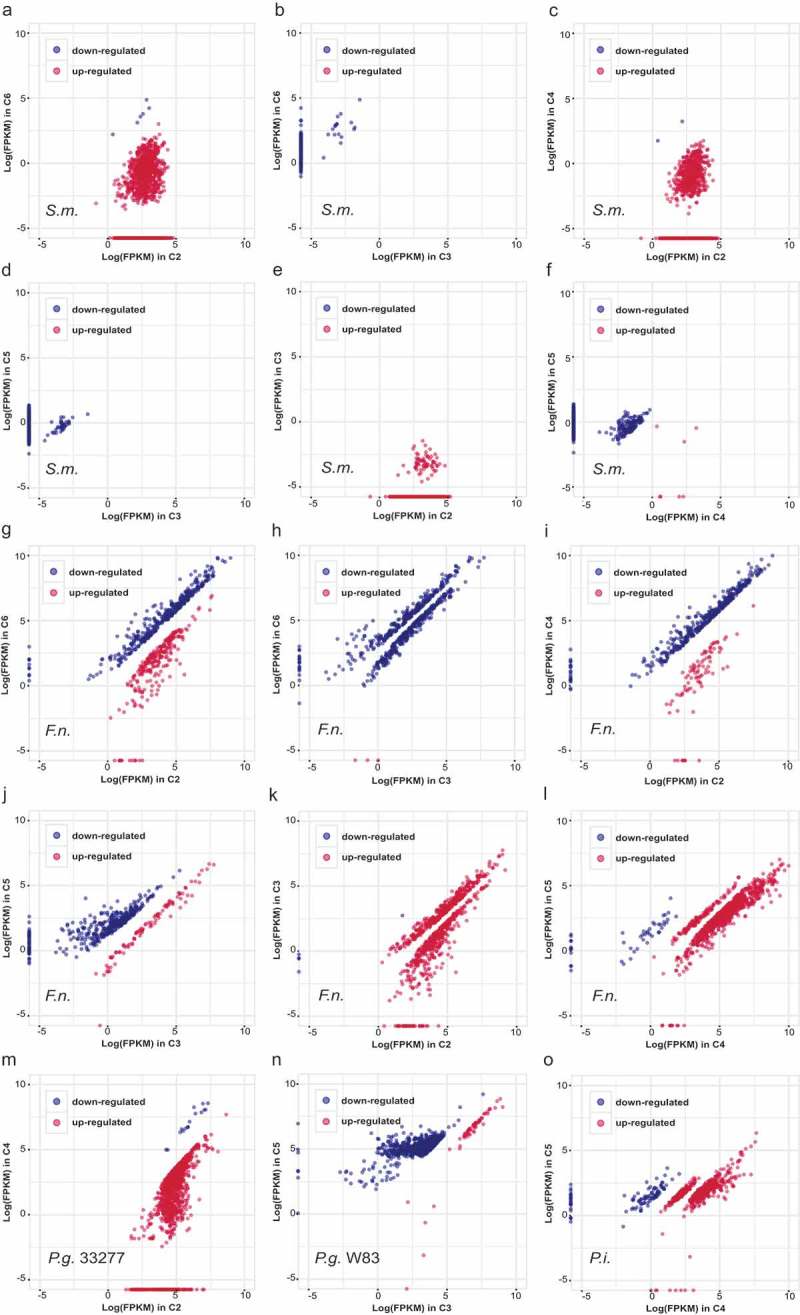
The significantly differential expressed genes (DEGs) of *S. mitis* (a-f), *F. nucleatum* (g-l), *P. gingivalis* ATCC33277 (m), *P. gingivalis* W83 (n) and *P. intermedia* (o) between communities. Blue spots represent higher expression genes in a community labeled on y-axis; while red spots represent higher expression genes in a community labeled on x-axis. Differences were considered significant at a *q*-value ≤0.05 and fold-change ≥2.

To further evaluate the effects of the ‘red’ and ‘orange’ complexes members on the gene expression of strains present in the commensal microbiota, community samples 2, 3, 4, 5, and 6 at day 9 demonstrating the most stable patterns, were subjected to metatranscriptomic sequencing. Compared to the non-pathogenic strain *P. nigrescens*, we observed significant expression alterations in *S. mitis* and *F. nucleatum* caused by both *P. gingivalis* ATCC33277 and W83. However, they had different effects. For example, *P. gingivalis* ATCC33277 altered the expression of 70% and 38% of *S. mitis* (C2 vs. C6, Figure 2(a)) and *F. nucleatum* (C2 vs. C6, Figure 2(g)) genes, respectively; roughly equal numbers of genes were up- and down-regulated. *P. gingivalis* W83 modulated the expression of 10% and 29.0% of *S. mitis* (C3 vs. C6, Figure 2(b)) and *F. nucleatum* genes (C3 vs. C6, Figure 2(h)), respectively, the majority of which were down-regulated. The presence of *P. intermedia* modified the alternations caused by *P. gingivalis* significantly (C4 vs. C2 and C5 vs. C3, Figure 2(c,d,i,j)), indicating that the interactions between community members could be easily disturbed by other organisms.

**Figure 3.**
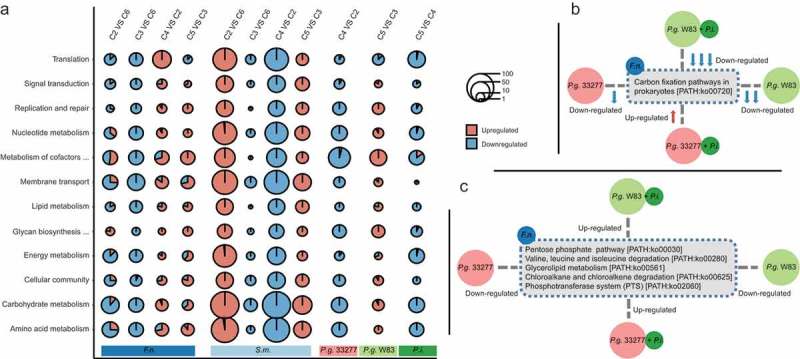
(a) The top 12 KEGG pathways of DEGs in *S. mitis, F. nucleatum, P. gingivalis* ATCC33277, *P. gingivalis* W83 and *P. intermedia* between communities (C2 vs C6; C3 vs C6; C4 vs C2; C5 vs C3; C5 vs C4). The y-axis shows the top 12 most abundant pathway categories. Red and blue colors represent up- and down-regulated genes, respectively. (b,c) Pathways that were specially altered in *F. nucleatum* by different strains. The upward arrows in red and downward arrows in blue represented up- and down-represented pathways or genes, respectively; the number of arrows represents the degree of change.

We next performed KEGG analysis of the differential expressed genes (DEGs) (Figure 3(a); Table S1-S6). Most DEGs in *F. nucleatum* that were down-regulated by *P. gingivalis* ATCC33277 and W83 belonged to the same KEGG pathway categories; except for some pathways that were only up-regulated by *P. gingivalis* ATCC33277, including steroid biosynthesis, thiamine metabolism, nicotinate and nicotinamide metabolism, phosphonate transport system permease protein phnE, and lipopolysaccharide export system permease protein lptG. In contrast, most *S. mitis* genes were up-regulated by *P. gingivalis* ATCC33277 but were down-regulated by W83, which mainly related to carbohydrate metabolism, nucleotide metabolism and membrane transport; reflecting differences in the regulation and response mechanisms of different bacteria. In C4 compared to C2, 380 *F. nucleatum* genes were up-regulated in the presence of *P. intermedia*. Of note, certain pathways that were altered by *P. intermedia* showed no significant changes between C2 and C6. These included ribosome proteins, vitamin B6 metabolism, zinc transport system permease protein znuB, manganese/zinc/iron transport system permease protein troC, troD, mntC, mntD, and the HIF-1 signaling pathway, while 1,249 *S. mitis* and 86 *F. nucleatum* genes were down-regulated. In C5 compared to C3, most DEGs in *F. nucleatum* and *S. mitis* were up-regulated by *P. intermedia*. The pathways that were particularly up-regulated in *F. nucleatum* by *P. intermedia* were those related to the pentose phosphate pathway, glyoxylate and dicarboxylate metabolism, nitrogen metabolism, porphyrin and chlorophyll metabolism, zinc transport system substrate-binding protein znuA, and iron complex transport system ATP-binding protein, while the pathway that related to bacterial chemotaxis were down-regulated.

**Figure 4.**
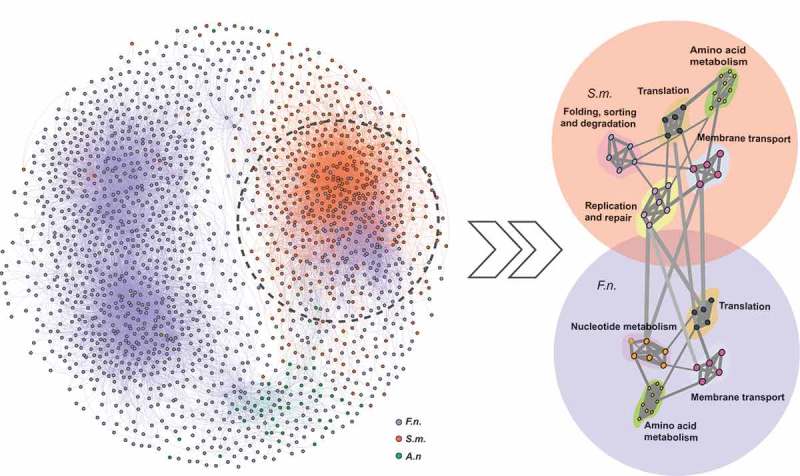
The gene-level interaction network among *F. nucleatum* (*F.n*. blue spots), *S. mitis* (*S.m*. red spots) and *A. naeslundii* (*A.n*. green spots). The schematic diagram depicts the most abundant gene functional categories involved in the *F. nucleatum-S. mitis* interaction (grey circle).

According to the results, we further identified some pathways that were specially modified in *F. nucleatum* (Figure 4(b,c)). Compared to *P. nigrescens*, the expression of the pathway relating to carbon fixation pathways in prokaryotes were down-regulated in *F. nucleatum* by *P. gingivalis* ATCC33277 (C2 vs. C6), and the down-regulation was more severe when co-cultured with *P. gingivalis* W83 (C3 vs. C2). Co-culture of *P. intermedia* and *P. gingivalis* W83 further aggravated the down-regulation (C5 vs. C3), while *P. intermedia* reversed the effect of *P. gingivalis* ATCC33277 (C4 vs. C2). Compared to *P. nigrescens*, both *P. gingivalis* ATCC33277 and W83 down-regulated the pathways related to the pentose phosphate pathway, valine, leucine and isoleucine degradation, glycerolipid metabolism, chloroalkane and chloroalkene degradation, and phosphotransferase system (PTS), while *P. intermedia* reversed the effect of both *P. gingivalis* ATCC33277 and W83. Accordingly, by affecting *P. gingivalis* (especially the W83 strain), *P. intermedia* may influence these metabolic pathways and further interfere with the proliferation of *F. nucleatum*.

## Interactions between *F. nucleatum* and *S. mitis*

Based on the gene expression of each strain in different communities, the correlations among the commensal microbiota at the gene-level were also accessed (Figure 4). The strongest interaction was between *F. nucleatum* and *S. mitis* (2,743 correlative links contributed by 416 *F. nucleatum* and 395 *S. mitis* genes [R ≥ 0.8]), followed by *F. nucleatum* and *A. naeslundii* (215 correlative links; between 91 *F. nucleatum* and 36 *A. naeslundii* genes [R ≥ 0.8]). The correlation between *S. mitis* and *A. naeslundii* was weakest (21 correlative links between 16 *S. mitis* genes and 9 *A. naeslundii* genes [R ≥ 0.8]). The correlated genes were classified into the Kyoto Encyclopedia of Genes and Genomes (KEGG) functional categories. The interaction between *F. nucleatum* and *S. mitis* consisted mainly of *F. nucleatum* genes belonging to the membrane transport, translation, amino acid metabolism, and nucleotide metabolism categories, and *S. mitis* genes belonging to the membrane transport, translation, amino acid metabolism, replication and repair, and folding, sorting, and degradation categories. The findings uncover the functional basis for the interactions between these two bacteria and the key role of *F. nucleatum* in biofilm formation.

**Figure 5.**
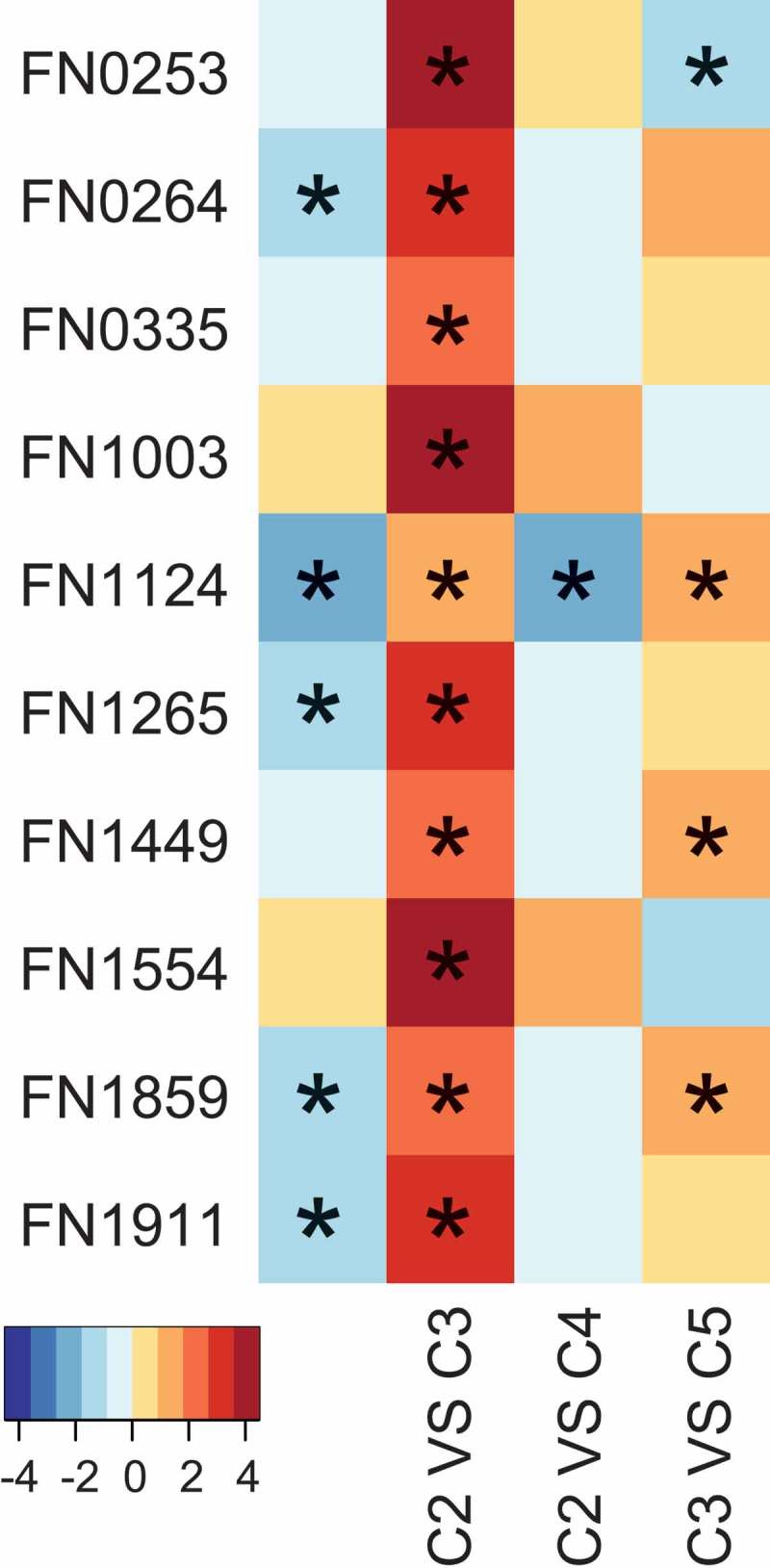
The differential expressed OMPs-related genes of *F. nucleatum* in each community. The color bar shows log2 fold change between two communities; * Differences were considered significant at a *q*-value ≤0.05 and fold-change ≥2.

Outer membrane proteins (OMPs) are involved in the molecular mechanisms that were utilized by *F. nucleatum* to physically bind to key members of the oral community [25]. The differentially expressed OMPs-related genes are listed in Figure 5. Compared to *P. nigrescens*, five OMPs – Fad A (FN 0264), FN1124, FN1265, FomA (FN1859) and FN1911 were down-regulated by *P. gingivalis* ATCC33277, and *P. gingivalis* W83 further aggravated their down-regulation. In addition to these, *P. gingivalis* W83 also down-regulated FN 0253, FN0335, FN1003, Fap2 (FN1449) and CmpA (FN1554) (C2 and C3). Compared to *P. gingivalis* ATCC33277, the addition of *P. intermedia* had almost no effect on the expression of OMPs, except for up-regulating FN1124 (C2 and C4). While compared to *P. gingivalis* W83, the addition of *P. intermedia* up-regulated FN0253 but down-regulated FN1124, Fap2 and FomA.

## Interactions between *P. intermedia* and keystone pathogens

The presence of *P. intermedia* (C4 and C2) modulated the expression of 55.9% of *P. gingivalis* genes; most of which were down-regulated. In C5 compared to C3, *P. intermedia* influenced the expression of 41.0% of *P. gingivalis* W83 genes, most of which were up-regulated. In C5 compared to C4, 35.2% of *P. intermedia* genes were down-regulated (Figure 2(m–o)). Though most genes were down-regulated in *P. gingivalis* ATCC33277, pathways that related to carbon fixation pathways in prokaryotes, foxO signaling pathway and ATP-dependent chaperone ClpB, were particularly up-regulated. As to *P. gingivalis* W83, the up-regulated pathways included sphingolipid metabolism, zinc transport system ATP-binding protein znuC; iron complex transport system, and lipopolysaccharide export systems lptB and lptG. In the presence of *P. intermedia, P. gingivalis* W83 showed higher expression of several putative virulence factors, including iron compound ABC transporter ATP-binding proteins, ferrous iron transporter, fimbrilin, capsular polysaccharide biosynthesis proteins, and hemolysin (Table S4 marked in red), which were down-regulated in *P. gingivalis* ATCC33277 (Table S3 marked in red). Compared to *P. gingivalis* ATCC33277, *P. gingivalis* W83 down-regulated the majority of *P. intermedia* genes related to purine metabolism, pyrimidine metabolism, glycolysis/gluconeogenesis, carbon fixation pathways, and ribosome (C4 and C5). However, the pathway related to arabinogalactan biosynthesis was up-regulated.

**Figure 6.**
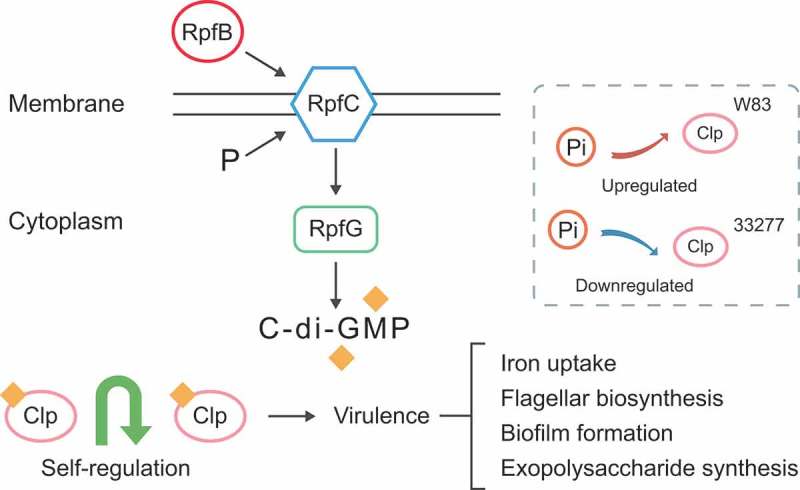
Model for *P. intermedia* regulating the expression of *P. gingivalis* virulence through the Clp regulator. Clp, a c-di-GMP effector that connects virulence to c-di-GMP modulation systems, i.e. RpfC/RpfG (pathway: KO 02024). The quorum sensing system may influence virulence gene expression through control of the intracellular level of Clp (crp, K10914). *P. intermedia* can upregulate the expression level of Clp in *P. gingivalis* W83, but down-regulates that in *P. gingivalis* ATCC33277.

Quorum sensing is a regulatory system that can control the biological processes of bacteria such as virulence, competence, conjugation, antibiotic production, motility, sporulation, and biofilm formation [26]. So, we evaluated the changes in the expression of quorum sensing-related genes of *P. gingivalis* ATCC33277 and *P. gingivalis* W83. As expected, *P. intermedia* up-regulated the expression of most QS-related genes in *P. gingivalis* W83, whereas it down-regulated the expression of QS-related genes in ATCC33277. It is notable that the genes related to *luxS* (K07173), *crp* (K10914), *ribD* (K11752) and *orpM* (K18139) were down-regulated in *P. gingivalis* ATCC33277 (Table S3 marked in green) but up-regulated in *P. gingivalis* W83 in the presence of *P. intermedia*. Cyclic AMP receptor protein (clp, K10914) is involved in iron uptake, multidrug resistance, and flagellar biosynthesis; and *ribD* (K11752) and *orpM* (K18139) are related to riboflavin metabolism (Table S4 marked in green). The effect of *P. intermedia* on the expression of QS-related genes in *P. gingivalis* was consistent with that on its virulence factor production. For example, genes related to iron transport and metabolism of cofactors and vitamins were up-regulated in *P. gingivalis* W83 but down-regulated in *P. gingivalis* ATCC33277 in the presence of *P. intermedia*, as were the QS-related genes *crp, ribD*, and *orpM*. Thus, we surmised that *P. intermedia* may modulate virulence factor production in *P. gingivalis* by regulating the expression of QS-related genes (Figure 6).

## Discussion

This work shed light on the functional regulation induced by cell–cell interactions within a complex community. Our study estimated the relative importance of individual and interactive effects of different complex members on the structural and functional dysbiosis of subgingival biofilm. Structurally, a dysbiotic community is characterized by a greater abundance of the red and orange complexes [11,27]. In this study, the results preliminarily indicated that common oral bacteria in biofilms may have strain-specific symbiotic relationships, and they will exhibit differential growth characteristics when they encounter different partner bacteria. When each commensal strain was cultured separately, we found that *S. mitis* grew faster than other commensal species. The same pattern existed in the mixed culture system C1, in which *S. mitis* made a dominating proportion on day 5. However, it did not apply to other groups (C2-C6), indicating other non-commensal species may depress *S. mitis*’ growth. We also confirmed that the keystone pathogen is crucial to cause the structural dysbiosis of the microbial community, and the high-virulence strain has a more significant impact. On the other hand, the effect of pathogenic bacteria on the microbiota structure is dynamic and may be related to the growth characteristics of different species: e.g. pioneer colonizers such as *S. mitis* can increase in numbers and proportions within hours after plaque removal [28]; the levels of orange complex species showed a sharp increase from days 4 to 7 [29]. In contrast, members of the red complex required more than 7 days to be established in the subgingival community [29]. From the results of the dynamic curve of each strain, we observed the greatest difference at day 5, which is generally consistent with previous reports. These findings suggested that taxonomic information may have less value if a time-dynamic factor is not considered.

During plaque biofilm formation, *S. mitis*, one of the major initial colonizers, would bind with *F. nucleatum*, which functions as a ‘bridge’ between early and later colonizers [30]. Our results suggested that *F. nucleatum* and *S. mitis* had a strong correlation at both species and gene level. At the species-level, the correlation may be mediated by the co-adherence between them. When the binding was interfered by other species or environmental factors, the correlation would be altered or even abolished. At the gene-level, the correlated genes between *S. mitis* and *F. nucleatum* were mainly related to the metabolism of secondary metabolites (such as amino acid and nucleotide), membrane transport and genetic information processing, indicating that they may not just contact with each other physically, but also have biological interplay. Gabor et al. [] found that the ratio of *S. mitis* and *F. nucleatum* are characteristically altered in the oral potentially malignant disorders lesions (OPMD) compared to the healthy mucosa. They inferred that the decreased relative abundance of *S. mitis* and an increased relative abundance of *F. nucleatum* might play a role in the transition of OPMDs to oral cancer [31]. However, the exact mechanism of their interactions needs to be further investigated.

To determine whether the addition of different bacteria can interfere with the interactions between community members, we investigated the expression of OMPs in *F. nucleatum*, including FN0253, FN0254, FadA (FN0264), FN0335, FN0387, FN0394, FN0517, FN1003, FN1124, FN1265，Fap2 (FN1449), RadD (FN1526), CmpA (FN1554), FomA (FN1859), FN1893, FN2047, and Aim1 (FN2058) [25,32,33]. These results showed that the involvement of different bacteria significantly affected the expression of the OMPs in *F. nucleatum*, indicating the co-adherence among *F. nucleatum*, and other partners have been changed when a new partner was added in. Among them, FadA and FomA exhibited the highest expression levels in each community. It has been reported that FomA is involved in binding between *F. nucleatum* and *P. gingivalis* in periodontal pockets [34]. Our results confirmed that the expression of *fom*A was changed when *F. nucleatum* was co-cultured with different *P. gingivalis* strains, indicating a strain-specific interplay between *F. nucleatum* and *P. gingivalis*. FadA (*Fusobacterium* adhesin A), is reported to be required for *F. nucleatum* attachment to epithelial cells and may play an important role in *Fusobacterium* colonization in the host [33]. Jung et al. reported that the invasion of *F. nucleatum* into gingival epithelial cells was suppressed in the presence of *P. gingivalis* [35]. Our results revealed that *P. gingivalis* could down-regulate the expression of *fad*A in *F. nucleatum*, which may be a possible explanation of their finding.

The presence of the ‘keystone’ pathogen *P. gingivalis* alters the profile of gene expression of the commensal community members significantly, which agrees with the reports of Frias-Lopez et al. [36]. However, the changes were specific depending on the strain used. For example, most genes in *S. mitis* were up-regulated by *P. gingivalis* strain ATCC33277 but down-regulated by W83. The addition of *P. intermedia* in the community altered the expression of virulence factors in *P. gingivalis*, and the regulation was also strain-specific. The *P. intermedia* strain isolated from a diseased periodontal site exerts synergistic effects with *P. gingivalis* W83 but antagonistic effects with strain ATCC33277. Barbosa *et al*. also reported that the outcome of the association between *P. gingivalis* and *P. intermedia* may be strain-dependent [37], which is consistent with our findings. It may explain why the keystone pathogen can be recovered from healthy sites but is not actively involved in disease.

Quorum sensing signals are used by bacteria to regulate biological functions. The expression of virulence factors by bacteria can be dependent upon quorum-sensing mechanisms [38]. Here we attempted to link the expression changes of QS related genes with the changes in virulence factors. Cyclic diguanylate monophosphate (c-di-GMP) is an important global signaling molecule that can regulate diverse bacterial physiological processes through its downstream receptors such as biofilm formation [39] and virulence [40,41]. However, c-di-GMP and c-di-AMP signaling have been rarely studied in oral biofilms [42]. Clp is a c-di-GMP effector acting as a positive transcription regulator in virulence gene expression [43,44]. We found that clp in *P. gingivalis* W83/33277 was up/down-regulated by *P. intermedia*. Targets virulence factors of clp, such as iron uptake was also up/down-regulated in *P. gingivalis* W83 and ATCC33277, respectively. Our working hypothesis is that *P. intermedia* may regulate the expression of virulence factors of *P. gingivalis* through the QS systems, which needs further verification.

In this study, we did not focus on the whole activities from the whole microbial communities but on individual functional changes resulting from the interaction between strains. Our results suggested that microbial interactions and keystone species within the indigenous microbiota have value in managing the subgingival microbial ecosystem. These data will also enable construction of gene-interaction networks and identification of key genes, which will enhance our understanding of the regulation of virulence factors and biofilm formation. However, one has to state, that this analysis based on *in vitro* grown biofilms with limited species and depended on the choice of clinical strains, which might be different from *in vivo* subgingival plaque that has been developed in the periodontal pocket.

## Data Availability

The authors declare that data supporting the findings of this study are available upon reasonable request.
