# Supplementary material for: Metatranscriptomic analysis of an in vitro biofilm model reveals strain-specific interactions among multiple bacterial species
Source: J Oral Microbiol. 2019 Apr 11;11(1):1599670. doi: 10.1080/20002297.2019.1599670 (PMC6461087; doi:10.1080/20002297.2019.1599670)
Supplement: Supplemental Material [file ZJOM_A_1599670_SM3409.zip › Supplemental files/supplemental(1).docx]

**Fig. S1** Dynamic curves depict the relative abundance of each strain changes over time in different communities (C1-C6).
